# Serological and Molecular Evidence of *Bartonella henselae* in Stray Cats from Southern Italy

**DOI:** 10.3390/microorganisms9050979

**Published:** 2021-04-30

**Authors:** Francesca Grippi, Paola Galluzzo, Annalisa Guercio, Valeria Blanda, Francesco Santangelo, Sonia Sciortino, Domenico Vicari, Francesca Arcuri, Santina Di Bella, Alessandra Torina

**Affiliations:** 1 Istituto Zooprofilattico Sperimentale della Sicilia “Adelmo Mirri”, via G. Marinuzzi 3, 90129 Palermo, Italy; francesca.grippi@izssicilia.it (F.G.); annalisa.guercio@izssicilia.it (A.G.); valeria.blanda@izssicilia.it (V.B.); sonia.sciortino@izssicilia.it (S.S.); domenico.vicari@izssicilia.it (D.V.); francescaarcuri11@gmail.com (F.A.); alessandra.torina@izssicilia.it (A.T.); 2 Dipartimento Scienze e Tecnologie Biologiche, Chimiche e Farmaceutiche, Viale delle Scienze, University of Palermo, 90133 Palermo, Italy; 3 U.O. Igiene Urbana ASP Palermo Presso Canile Municipale, Piazza Tiro a Segno 5, 90141 Palermo, Italy; franco.santangelovet@gmail.com

**Keywords:** *Bartonella henselae*, cats, seroprevalence, real-time PCR

## Abstract

*Bartonella henselae* is a slow growing and facultative intracellular pathogen mainly transmitted by arthropod vectors adapted to domestic and wild mammalian reservoir hosts. Since cats are the major source of the *B. henselae* infection, this study aimed to evaluate the seroprevalence and the DNA presence in randomly sampled stray cats. Blood samples of 429 cats were collected from shelter of Palermo (Southern Italy) and sera and whole blood were analyzed for the presence of antibodies against *B. henselae* by indirect immunofluorescence assay (IFA) and by real-time polymerase chain reaction (PCR), respectively. Two hundred and three sera (47.3%) were positive to IFA and 148 blood samples (34.5%) to real-time PCR. Based on serological results, the evaluation of the potential risk factors (sex, age, coat color) was carried out. The multivariate analysis indicated that cats more than 12 months old were more likely to be seropositive to *B. henselae* than cats <12 months. These data will add useful information to the understanding of the spread of *B. henselae* in stray cats in Southern Italy.

## 1. Introduction

*Bartonella* species are fastidious, slow growing and facultative intracellular pathogens mainly transmitted by arthropod vectors that are highly adapted to a wide range of domestic and wild mammalian reservoir hosts [1,2]. Domestic cats (*Felis catus*) are the primary reservoir of *B. henselae,* the main causative agent of the cat scratch disease (CSD). The CSD is a common zoonosis with a worldwide distribution, a benign lymphadenopathy characterized by local forms affecting the skin and the loco-regional lymph nodes [3,4]. The infection is usually benign and self-limiting, but sometimes, can evolve in systemic granulomatous forms, which acquire severity in immunocompromised hosts. The most frequent route of transmission to humans is via cat bites or scratches [5].

*Bartonella* infection in most cats is not usually characterized by clinical manifestations, but sometimes, diseased cats show self-limiting febrile illness, lymphadenopathy, transient anemia and neurological dysfunction [6]. Infected cats can remain bacteremic for long periods, even more than a year. Persistent bacteremia in asymptomatic cats represents the most important factor that facilitates the spread of the microorganism [7].

*B. henselae* is transmitted among cats mainly by the cat flea (*Ctenocephalides felis*), and this vector is essential for maintaining the infection within the cat population. *B. henselae* can multiply in the digestive system of the cat flea and survive at least 3 days in the flea feces. Therefore, the source of infections appears to be the flea feces that are inoculated by contaminated cat claws [7].

Previous epidemiological studies worldwide have shown a high level of variability of *B. heanselae* seroprevalence among cat populations in different geographic regions, and among individuals living in the same geographic area [7]. Seroprevalence of 1–8% in Germany and in Switzerland [8,9,10], 41% in France [11], 56% in Netherlands [12], 81% in California were reported [13]. Bacteremic prevalence rate often approach 50–75% in feral cat populations worldwide [14]. The differences in serological and bacteremic prevalence are related to different climatic conditions increasing from cold climate to warm humid climates. Some factors such as age, animal status (pet or stray) as well as geographic and environmental conditions might influence the risk of *B. henselae* infection. Stray cats roam freely and often form colonies that live and feed in close proximity to humans, so stray cats are potential sentinels, mainly in urbanized areas, for both human and pet health [15,16,17,18].

Regarding *Bartonella* seroprevalence in cats in terms of sex, there are contradictory results. According to some authors, no significant differences have been detected between the sexes [19,20,21]. However, some researchers [12,22] have reported that male cats had higher infection rates having more opportunities to be scratched or bitten by other cats while protecting their territories, whereas Sander et al. found that female cats had a higher prevalence [23]. Moreover, as occurs in other vector-borne diseases, a correlation between seroprevalence for *Bartonella* and coat color could be hypothesized. It is more difficult to detect parasites in dark hair than in animals with fair hair color, and this may delay anti-parasite interventions. This would allow more time for the pathogen to move from the parasites to the host and infect it [24].

Since vector-borne diseases can have an impact in both animal and human health, it is relevant for infectious disease control and mitigation strategies to identify the pathogen circulation in a certain geographic area. Moreover, since cats are the major source of the *B. henselae* infection, this study aims to evaluate the epidemiology of this bacterium by both molecular and serological approaches in stray cats living in Palermo (Sicily, Southern Italy). We hypothesized that the seropositivity to *Bartonella henselae* in urban free-ranging cats could be influenced by possible risk factors as age, sex and coat color.

## 2. Materials and Methods

### 2.1. Study Design and Specimens

During the period January–September 2018, 429 blood samples were collected into serum and Ethylenediaminetetraacetic acid (EDTA) tubes from censused stray European race cats belonging to registered colonies of Palermo (Sicily, Italy). The sample size was determined using Winepi software considering an expected prevalence of 50%, with 5% precision at the 95% confidence level (as no other epidemiological data were available). According to Winepi, the minimum number of required samples was 270 animals. The cats were randomly chosen among those that arrived at the shelter to be spayed or neutered and then released into the environment and reintroduced in their colony. Veterinarians reported no overt symptoms of bartonellosis in the cats that were considered clinically healthy.

### 2.2. Serological Test

About of 1 mL of blood were collected into a 3 mL tube containing no anticoagulant, stored in a refrigerated bag and conferred to the laboratories of the Istituto Zooprofilattico Sperimentale of Sicily.

All samples were centrifuged at 1500× *g* for 15 min and then serum was separated from the clot and the sera were collected and immediately tested or stored at −20 °C. Antibodies to *B. henselae* were detected by the commercial test MegaFLUO^®^ BARTONELLA henselae IFA IgG (MEGACOR Diagnostik GmbH, Horbranz, Austria) according to the manufacturer’s instructions. All sera were diluted 1:50 and 1:100 with PBS as suggested by the manufactures. The results were visualized using standard fluorescence microscopy, where a positive reaction was seen as sharply defined yellow-green fluorescent inside infected cells. A negative reaction was seen as the absence of sharply defined proteobacteria, like that seen in the negative control well. Positive and negative controls were supplied in the kit. All sera with titers ≥1:50 were considered positive [25].

### 2.3. DNA Extraction and Real-Time Polymerase Chain Reaction (PCR)

Whole blood into an EDTA tube was collected and used to extract DNA. The DNA was extracted using the commercial kitMagMAX CORE (Thermo Fisher Scientific, Monza, Italy), following the manufacturer’s protocol, and stored at −20 °C. DNA samples had an average concentration between 9 and 32 ng/µL and an A260/280 ratio between 1.8 and 2. A screening on blood samples was carried out by real-time PCR in order to check for the presence of *B. henselae* DNA. The real-time PCR protocol proposed by Alamán Valtierra in 2016 was used [26], but a qualitative rather than a quantitative approach was carried out. Real-time PCR was performed using the CFX96 Touch Real-Time PCR Detection System (Bio-Rad, Hercules, CA, USA), amplifying a 183-base pair product of the specific 16S-23S intergenic region of *B. henselae* ribosomal RNA [26]. DNA was added to a reaction mix containing 1× of SSo advanced Universal Sybr Green Supermix (Bio-Rad, Hercules, CA, USA) and 0.5 µL of each primer (10 µM) (Table 1) up to a final volume of 25 µL. The instrument program consisted of an initial denaturation of 3 min at 95 °C followed by 40 cycles of amplification, which included a denaturation step at 95 °C for 10 s and an annealing/extension step at 60 °C for 30 s. The final step consisted in 0.5 °C increment from 65 to 95 °C for 2 s/step to analyze the melting curve. All runs included a field sample positive to *B. henselae* and a negative (DNAase/RNAase free water) control.

### 2.4. Statistical Analysis

The statistical analysis was performed by multiple logistic regression models. The significant variables were identified using the maximum likelihood estimates. To assess the risk factors, corresponding *p*-values and odds ratios (OR) for the examined variables were evaluated, with a 95% confidence interval (95% CI). The mathematical modelling was performed using the SAS/STAT software version 9.4 (SAS Institute Inc., Cary, NC, USA).

The cats were compared with respect to age (kitten ≤12 months and adult >12 months), sex (male or female) and coat color (uniform or mixed color). The cut off points were selected considering average characteristics of the analyzed cats.

### 2.5. Ethical Statement

The study did not involve any animal experiment. Only sample collection from naturally infected cats was carried out consisting of a single blood draw per cat. This was needed for the laboratory analyses and did not involve any suffering of the sampled animals. This study was conducted as part of the IZS SI 11/16 RC research project entitled “Indagine sulla diffusione di Borreliosi e Bartonellosi in canili del territorio regionale e potenziali rischi zoonosici correlati” approved by the Italian Ministry of Health on 9 August 2017.

## 3. Results

### 3.1. Animal Demographics

A total of 429 stray cats were sampled (166 male and 263 female). The cats were divided into two groups based on age 215 kittens (<12 months) and 214 adults (>12 months). In relation to the coat color, 297 cats had a mixed color coat and 132 a uniform color coat.

### 3.2. Serological Tests

Out of 429 sera samples, 203 had antibodies against *B. henselae*, 24 with a titer of 1:50, 179 with titer of 1:100 (Table 2). The overall seroprevalence was 47.3%.

### 3.3. Risk Factors Analysis

The results obtained were examined by the multivariate analysis in order to determine the correlation, if any, between IFA results and three different variables: age, sex and coat color (Table 3). The multivariate analysis showed that only age variable was significantly related to *B. henselae* seropositivity (χ^2^ = 4.7004; *p* < 0.0302). Adult cats were about one and a half times more likely to be seropositive compared to kittens. In fact, the seroprevalence in adult cats was 53.3% while in kittens it was 41.4%. No correlation was found for other variables.

### 3.4. Molecular Analysis

The real-time PCRs mean values of efficiency = 95.6 ± 2.6, slope = −3.4328 ± 0.07, R^2^ = 0.998 ± 0.003, and y-intercept = 34.049 ± 0.31 were evaluated. Real-time PCR carried out in the whole blood of all the 429 cats revealed that 148 cats (34.5%) contained *B. henselae* DNA. The positive control corresponded to a Tm of 81, while positive samples corresponded to a mean tm of 79.66 ± 0.83.

The real-time PCR and serological results are reported in Table 4.

## 4. Discussion

*Bartonella henselae* infection is common in stray and domestic cats and it is known that cats remain a reservoir of the zoonotic bacteria. Cats are ubiquitous and often in intimate contact with potential susceptible humans. Since infected cats usually remain asymptomatic, close contact with stray and wild cats may present a risk of acquiring *Bartonella* infection for humans and animals. Transmission from cats to humans occurs via a scratch or bite and is due to the presence of the bacterium on claws and in the oral cavity. Flea-infested cats tend to have higher grooming activity than non-infested cats. These circumstances would make a bacteremic cat a major risk for humans [27].

In this study the presence of anti-*B. henselae* antibodies and the DNA of *B. henselae*, in blood sample of stray cats from Palermo (Southern Italy) was assessed, in order to evaluate the prevalence of this pathogen. Cats captured from January to September 2018 were free-roaming and inhabiting urban streets, they did not belong to a specific household and most of them were untamed. Cats harboured fleas at the time of blood collection. As they probably did not receive any preventive flea treatment they were significantly more likely to be infected, suggesting that vector-control programs are crucial to avoid *Bartonella* infection. Anti *B. henselae* antibodies were detected in 203 (47.3%) of 429 sera examined. Our results are in agreement with data reported for Northern Italy (45.9%) [28] and for Southern Italy (45.7%) [29].

The statistical analysis conducted on the serological data of cats has shown that age has proved to be a risk factor, showing that animals older than one year of life are more likely to become infected with *Bartonella henselae* than younger cats as previously reported by Fabbi et al. [27]. No associations between seropositivity, sex, and coat color was found. This study corroborates the findings of previous studies conducted in the same geographical area which have shown that the seroprevalence of *B. henselae* in cats is generally high [30,31]. Moreover, Mansueto et al. demonstrated the correlation between humans and stray cats seroprevalence, rather than pet cats in the study area, probably due to the lower environmental exposure to *Bartonella* and the better health state and medical surveillance of pet cats, compared to stray cats [30].

On all whole blood samples, the DNA of *B. henselae* was searched by real-time PCR. Given the large number of samples, the real-time PCR proposed by Alamán Valtierra in 2016 [26] was converted into a qualitative PCR. In this way it was possible to carry out a rather rapid screening using Sybr green real-time PCR reducing costs compared to a Taqman probe-based real-time PCR.

*B. henselae* DNA was detected in 148 (34.5%) of 429 cats. *B. henselae* DNA was previously detected in the 27.1% and in the 20.9% of stray cats blood sample in Northern Italy and in Southern Italy respectively [28,29]. Other studies report finding *B. henselae* DNA in 56.9% of blood samples from 51 wild cats from Alabama and Florida [32] and in 41.8% of blood samples from 150 wild cats in Korea [33].

These studies reveal the variability of the results in the different geographical regions depending on the origin of the cats.

Out of 203 cats serologically positive for *B. henselae*, 117 were negative for the presence of pathogen DNA in the blood. These subjects were probably no longer in the phase of bacteremia and it can be assumed that the sample was sampled in a time distant from the infection. Moreover, 20 of the 117 cats have an antibody titer of 1:50 (low titer) and 97 cats had an antibody titer ≥1:100 (high titer). Most likely, the antibody titer of 1:50 corresponds to a remission stage of *B. henselae* infection, more distant from infection than in cats with an antibody titer ≥1:100.

Maggi and colleagues [34] developed a culture medium (*Bartonella*/alpha-Proteobacteria growth media, BAPGM) to support the growth of at least seven *Bartonella* species and to facilitate the isolation of *B. henselae* from blood and fluids. The molecular diagnosis of *B. henselae* was reported to be improved by pre-enrichment in BAPGM [35]. The lack of this enrichment phase in our study could be the basis of the discrepancy between negative real-time PCR samples from seropositive cats, although it is unlikely to account for all cases. Additionally, treatment histories were unknown, and previous antibiotic treatment may have adversely affected PCR DNA detection in *Bartonella* seropositive cats.

Among the positives for the presence of *Bartonella* DNA in the blood, 62 were seronegative and 86 seropositive for *B. henselae*.

The antibody response to *B. henselae* has been demonstrated to occur 2 weeks after infectious challenge [36]. As cats can remain bacteremic for up to 32 weeks [37], the presence of both bacteremia and elevated antibody titers is a common feature. This result indicates that the 62 cats were in the early or acute phase of infection and therefore IgM rather than IgG antibodies may have been present. Therefore, these cats could be carriers of the pathogen and so they could be contagious.

Determination of the absence of bacteremia is crucial in assessing actual risk of transmission for the cat; however, a non-bacteremic cat with positive serology results should be re-evaluated for possible recurrent bacteremia [27].

This work takes interest in animal and human health. The infected cats in this study did not show clinical changes although it is possible that they had transient anemia associated with bacteremia that just was not detected during this study cause blood tests were not performed. Since animals included in this study were stray and animals that may be adopted from both urban and rural areas, routine procedures of detection in shelters should be carried out, as well as effective vector control, in order to reduce the spread of the pathogens and avoid health problems in both pets and their owners. Furthermore, we emphasize the importance of managing flea control in the adopted cats and the environment and avoiding scratches and cat bites due to the potential risk that *Bartonella* infection represents mainly in immunosuppressed people.

## Figures and Tables

**Table 1:** Nucleotide sequence of primers used to reveal *B. henselae* DNA by real-time PCR assay.

Name	Sequences 5′-3′	Gene	Amplicons	Reference
*B_hen F*	TGTCATCAGAAAGGGCTATT	16S-23S	183 bp	[26]
*B_hen R*	CAAAACAAAGTGCAAAACAA			

**Table 2:** Distribution of *Bartonella henselae* different seropositivity (1:50 and 1:100).

Factors	Titer 1:50	Titer 1:100
**Age**		
<12 months	16	73
>12 months	8	106
**Sex**		
Female	14	108
Male	10	71
**Coat color**		
Mixed color	16	131
Uniform color	8	48

**Table 3:** *Bartonella henselae* seropositivity according to characteristics of cats: percentage of positive, number of positive, odds ratio related 95% confidence intervals (95% CI); reference (Ref.).

Factors	% pos	N° pos/All	OR (95% CI)
**Age**			
<12 months	41.4	89/215	Ref.
>12 months	53.3	114/214	1.593 (1.046–2.426)
**Sex**			
Female	46.4	122/263	Ref.
Male	48.8	81/166	1.045 (0.679–1.608)
**Coat color**			
Mixed color	49.4	147/297	Ref.
Uniform color	42.4	56/132	0.736 (0.466–1.161)

**Table 4:** Real-time PCR and seropositivity results in 429 cats.

Status of Cat	Real-Time PCR Results		
	Positive (%)	Negative (%)	Total (%)
Seropositive	86 (20)	117 (27.3)	203 (47.3)
Seronegative	62 (14.5)	164 (38.2)	226 (52.7)
**Total**	148 (34.5)	281 (65.5)	429

## Data Availability

Data are contained within the article.
